# Shoe leather epidemiology: active travel and transport infrastructure in the urban landscape

**DOI:** 10.1186/1479-5868-7-43

**Published:** 2010-05-11

**Authors:** David Ogilvie, Richard Mitchell, Nanette Mutrie, Mark Petticrew, Stephen Platt

**Affiliations:** 1 Medical Research Council Epidemiology Unit and Centre for Diet and Activity Research (CEDAR), Cambridge, UK; 2 Section of Public Health and Health Policy, University of Glasgow, Glasgow, UK; 3 Department of Sport, Culture and the Arts, University of Strathclyde, Glasgow, UK; 4 London School of Hygiene and Tropical Medicine, London, UK; 5 Centre for Population Health Sciences, University of Edinburgh, Edinburgh, UK

## Abstract

**Background:**

Building new transport infrastructure could help to promote changes in patterns of mobility, physical activity, and other determinants of population health such as economic development. However, local residents may not share planners' goals or assumptions about the benefits of such interventions. A particularly contentious example is the construction of major roads close to deprived residential areas. We report the qualitative findings of the baseline phase of a longitudinal mixed-method study of a new urban section of the M74 motorway in Glasgow, Scotland, that aims to combine quantitative epidemiological and spatial data with qualitative interview data from local residents.

**Methods:**

We interviewed 12 residents purposively sampled from a larger study cohort of 1322 to include men and women, different age groups, and people with and without cars, all living within 400 metres of the proposed route of the new motorway. We elicited their views and experiences of the local urban environment and the likely impact of the new motorway using a topic guide based on seven key environmental constructs (aesthetics, green space, convenience of routes, access to amenities, traffic, road danger and personal danger) reflecting an overall ecological model of walking and cycling.

**Results:**

Traffic was widely perceived to be heavy despite a low local level of car ownership. Few people cycled, and cycling on the roads was widely perceived to be dangerous for both adults and children. Views about the likely impacts of the new motorway on traffic congestion, pollution and the pleasantness of the local environment were polarised. A new motorway has potential to cause inequitable psychological or physical severance of routes to local amenities, and people may not necessarily use local walking routes or destinations such as parks and shops if these are considered undesirable, unsafe or 'not for us'. Public transport may have the potential to promote or discourage active travel in different socioeconomic contexts.

**Conclusions:**

Altering the urban landscape may influence walking and cycling in ways that vary between individuals, may be inequitable, and may not be predictable from quantitative data alone. A more applied ecological behavioural model may be required to capture these effects.

## Background

### Active travel and the urban landscape

Walking and cycling as modes of transport ('active travel') can make an important contribution to overall physical activity and may be associated with characteristics of the built or natural environment [1-3]. *Prima facie*, altering the urban landscape may lead to changes in patterns of mobility, physical activity and (eventually) population health. These changes may be positive or negative, and may occur as the indirect or unintended effect of transport or planning policies primarily intended to achieve other goals [4].

This plausible line of reasoning requires two important caveats. First, the putative causal chain linking changes in the physical environment to changes in patterns of health-related behaviour has rarely been empirically tested in robust longitudinal studies [5]. Second, most studies of the environmental correlates of physical activity have been conducted in North America and Australia, typically in settings with socioeconomic and environmental characteristics not necessarily found elsewhere [6]. Hypotheses about the effects of altering the urban landscape should therefore be tested more rigorously and in a wider range of settings.

### Social inequalities in mobility and its impact

In the UK, where access to a car is strongly associated with household income, people without cars make fewer trips, spend less time travelling and cover less distance overall than those with cars, but travel 50% further on foot [7]. One obvious implication is that less-affluent groups may be disadvantaged in terms of their overall mobility, but may gain the benefit of additional physical activity through active travel as a result. A population shift towards this pattern of mobility offers a potentially winning combination of an increase in physical activity coupled with reductions in traffic congestion and use of fossil fuels, and is therefore generally regarded as desirable on public health, transport, environmental and equity grounds [8,9]. However, this view is not necessarily held by residents of deprived communities, who may not share planners' goals or assumptions about the benefits of new infrastructure [10], may experience having to walk through neglected surroundings as stressful [11], or may aspire to the protection, autonomy and prestige afforded by cars [12].

A particularly contentious type of intervention in the urban landscape is the construction of new major roads, which are sometimes routed through or near deprived residential areas [13]. In a systematic review, Egan and colleagues found no evidence about the effects of new major urban roads on physical activity or social inequalities in health, and little evidence to support a frequently-cited justification for such roads, namely that they reduce the incidence of injuries [14]. However, this review did find that new major urban roads were associated with increased disturbance from traffic noise [15-18], as well as with severance effects whereby residents' perceived boundaries of their own neighbourhoods were constrained and altered when their local areas were bisected by new roads [19-21]. These findings reflect those of the seminal study of Appleyard and Lintell of three streets in San Francisco, in which 'All aspects of perceived liveability [...] were found to correlate inversely with traffic intensity', including the size of residents' 'home territories' [22].

### The M74 motorway project in Glasgow

The construction of an urban motorway network in Glasgow, the largest city in Scotland, dates back to the 1960s and has involved the disruption, bisection or demolition of a number of established, mostly deprived, residential areas [13,23]. A new section of the M74 motorway is now to be added to the network at a cost of £457 million. It is claimed that the new motorway will relieve congestion, improve conditions for pedestrians and cyclists by reducing traffic on local streets, reduce traffic noise and bring new local employment opportunities, helping to regenerate some of the most deprived and least healthy urban communities in Europe (Table 1) [24]. Objectors claim that the new motorway will encourage car use, degrade the aesthetic quality of the surroundings and reduce the safety and attractiveness of routes for pedestrians and cyclists. An independent public local inquiry found that the new motorway 'would be very likely to have very serious undesirable results', notably in terms of its impact on local communities, and recommended against the proposal [25]. This advice having been overruled by the government [26], construction is now under way and expected to be completed in 2011.

**Table 1:** Claims related to health and wellbeing made for and against the new motorway

Domain*	Claims made in favour of intervention^†^	Claims made against intervention^†^
Economic	Will create up to 20,000 jobs by enabling regeneration and encouraging inward investment	Will redistribute economic activity from other parts of Scotland rather than producing a net increase
	Will increase business competitiveness by improving just-in-time delivery times	Will displace 100 local businesses
	Will create 350 jobs during construction	
Traffic	Will reduce journey times, relieve congestion on existing motorways and main roads, and reduce traffic on local roads	Will increase traffic in general and on feeder roads in particular
Injuries	Will reduce accidents	
Active travel	Quieter local roads will lead to improved conditions for pedestrians, cyclists and public transport	Will encourage use of motor vehicles Local walking and cycling journeys will be made more difficult by having to cross new motorway junctions
Environmental	Noise and air pollution will be reduced on balance throughout the area	Moderate-to-major increases in noise are predicted at some sites
	Will produce minimal severance effects because much of the route follows an existing main line railway	Nitrogen dioxide concentrations will be increased within 100 metres of the route
	Chromium-contaminated land will be handled safely during construction	Very severe combined impacts predicted in four residential areas close to the route
		Chromium will be dispersed from contaminated land into the air or river during construction
		Contradicts stated overall sustainability objectives of transport policy
Social justice	Will improve quality of life in local communities	Unacceptable opportunity cost, e.g. the money could be used to fund improved public transport
	Will result in better employment opportunities for local people	Will mostly benefit motorists from more distant and more affluent areas, causing adverse effects on local communities which have low levels of car ownership

* Claims grouped into domains *post hoc *by authors.

† Summarised and adapted from the then government's case for the project [24] and the report of the public local inquiry. [25]

We established a longitudinal observational study in the local population, the rationale and design for which have been described previously [4]. Rather than attempting to examine impacts across all possible domains of health and wellbeing, we chose to focus on the effects of the intervention on perceptions of the urban environment and patterns of active travel and physical activity. We framed the baseline study as a cross-sectional study in its own right, designed to explore the relationships between travel behaviour, perceptions of the urban environment, physical activity and socioeconomic position, as well as to inform the development of the follow-up study. Recognising the complex social, economic, political and environmental contextual factors and causal relationships involved [27], we adopted a mixed-method approach, aiming to combine the insights from quantitative epidemiological and spatial analysis with those from face-to-face qualitative interviews with local residents *in situ*. The principal findings of the baseline quantitative research have been reported previously [28,29]. The aim of this paper is to report the findings of the baseline qualitative research, to integrate them with the quantitative findings, and to reflect on the unique and interactive contributions of the two elements of the mixed-method approach. In homage to a classical form of community public health investigation, we refer to the qualitative study as 'shoe leather epidemiology'; not only was walking a major theme of the study, but all the fieldwork was conducted on foot or by bicycle as a means of immersion in the study environment.

## Methods

### Main survey

The methods of sampling and data collection have been described more fully elsewhere [29]. Briefly, we delineated three matching study areas on the basis of spatial and aggregate socioeconomic characteristics and surveyed adults in a random sample of households in these areas using a postal questionnaire that included items on demographic and socioeconomic characteristics, health and wellbeing, perceptions of the local environment, travel behaviour and physical activity. Respondents were also invited to return an 'opt-in' consent form allowing us to approach them for a follow-up interview.

### Interview study

#### Recruitment

**Figure 1:**
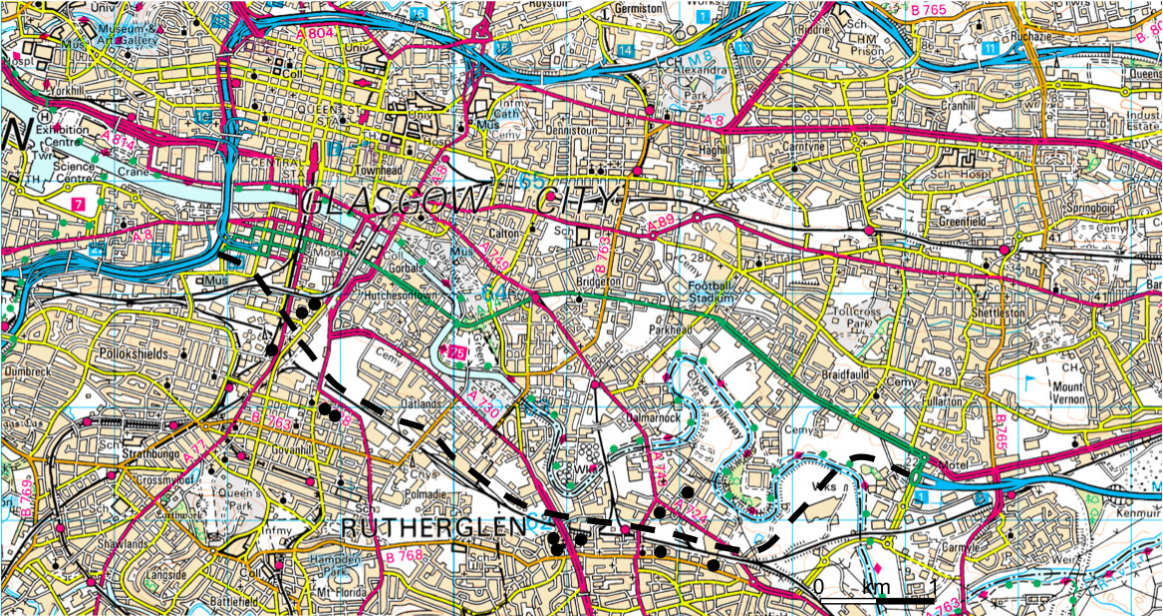
**Interview study area**. Dashed line indicates approximate proposed route of new motorway. Circles indicate approximate location of participants' homes. Raster image ^© ^Crown Copyright/database right 2005. An Ordnance Survey/EDINA supplied service.

From the report of the environmental impact assessment for the motorway proposal [30] we identified four neighbourhoods where particular positive or negative impacts of the motorway were predicted and where no major concurrent regeneration project was in progress (Table 2, Figure 1). We recruited interview participants purposively and iteratively by issuing invitations by letter or telephone in batches to consenting survey respondents living in these neighbourhoods, gradually assembling a sample that comprised a mixture of men and women, different age groups, people with and without access to a car, and people living in the different neighbourhoods. Participants were offered £10 as a token of appreciation for giving up their time for an interview. As in the main survey, the study was described to potential participants as being about 'traffic and health in Glasgow'. The motorway was not mentioned in any recruitment material, and the stated aim of the interviews was 'to help us understand better what it is like to live in your local area'.

**Table 2:** Neighbourhoods represented in the interview study

Neighbourhood	Characteristics	Participant numbers
Laurieston and Eglinton	A busy, noisy urban environment containing two major arterial roads, which the new motorway will cross on viaducts very close to some residential properties	P1, P11, P12
North east Govanhill	Close to feeder roads for a new motorway junction, but adjacent to the only section of the route which will run in a cutting	P4, P6
Rutherglen	A town centre in its own right with a mixture of traditional and modern housing close to the route, whose main street is predicted to experience substantial traffic reduction after the motorway opens	P2, P5, P7, P9, P10
Farme Cross	A satellite of Rutherglen on the north side of the route with a new, comparatively affluent private housing development which will be close to a new motorway junction	P3, P8

### Interviews

Interviews took place between February and June 2006, before the onset of motorway construction, and were conducted one-to-one in participants' homes except, in one case, in a local café at the participant's request. Each interview lasted for 30 to 60 minutes, was semi-structured using a topic guide (Table 3), and was subsequently transcribed from a digital audio recording made with the participant's consent.

**Table 3:** Topic guide for interviews

Theme	Prompts
Introduction	Explain purpose of research project
	Explain audio recording procedures
	Ensure participant has copy of information sheet
	Complete both copies of consent form
	Offer £10 for participating
Review questionnaire data	
Mapping task	Mark home
	Name local area and discuss boundaries
	Identify locations of key local amenities (shops, school, park, health centre...)
	Discuss routes for typical local journeys (and whether made on foot, by car...)
Living in the area	What do you like about living in the local area?
	What do you not like about living in the local area?
	What do you think of this area as a place to bring up children?
	Is there anything you would like to change about the local area?
	Environmental themes to be used as prompts if necessary:
	• Aesthetics (pleasant to walk, attractive surroundings...)
	• Green space (parks, in general...)
	• Convenience (of routes for walking and cycling)
	• Access to amenities (shops, public transport...)
	• Traffic (quantity, disturbance...)
	• Road danger (for pedestrians and cyclists)
	• Personal danger (of attack, after dark...)
M74	Do you know about the plan to build the new motorway?
	Explain briefly if necessary
	How do you think that will affect your local area?
	How do you think that will affect you and your household?
Close	Thanks for participating

Each interview began with a brief discussion of the participant's questionnaire responses to confirm basic details about themselves, their household circumstances and their activities. In early interviews in the series, a printed large-scale map was used to prompt discussion of participants' local areas, the location of key amenities and the routes of their typical journeys. The rest of the interview focused on exploring participants' perceptions and experiences of the area as a place in which to live and travel and how these might change as a result of motorway construction.

In the absence of any single satisfactory theoretical framework for conceptualising the influence of the environment on health-related behaviour [2], our approach was based on an ecological model of behaviour capable of encompassing people's transactions with their physical and sociocultural surroundings [31]; such models are increasingly popular and useful in studies of environmental influences on travel behaviour and physical activity, for example as described by Saelens and colleagues with respect to the specific behaviours of walking and cycling [1]. From a review of this body of literature [32], we identified *a priori *seven environmental constructs that were likely to be related to physical activity in general or walking and cycling in particular and that could reasonably be expected to change as a result of the intervention: aesthetics, green space, convenience of routes, access to amenities, traffic, road danger and personal danger. We used these constructs as prompts (where necessary) to elicit and organise participants' views in the interviews, and we elicited perceptions related to the same constructs using quantitative scales in our main baseline survey [28].

The topic guide was used flexibly, and since using the map appeared to contribute little to the discussion it was dispensed with in later interviews. However, one principle was rigidly adhered to: that the topic of the new motorway would not be introduced by the interviewer until the latter part of the interview, at which point he would ask the participant what effects, if any, they thought the motorway would have on their local area and their own situation.

Field notes were written immediately after each interview.

#### Analysis

The transcripts were checked against the audio recordings. An iterative process of analysis was then used to code segments of transcripts, extract related segments, identify and group themes, and identify patterns and negative cases using the method of constant comparison. The coding of segments and the identification of themes were non-exclusive, such that one excerpt of talk might be categorised under more than one theme. To begin with, higher-order themes were mostly derived from the topic guide, in that views about the local area and about the potential effects of the new motorway were initially grouped under the seven *a priori *environmental constructs. The lower-order themes emerged from the data elicited in the interviews; most could meaningfully be grouped under one of the higher-order themes, but a few spanned more than one higher-order theme or were not closely related to any of the *a priori *constructs. Overarching themes developed during the later stages of analysis tended to span various combinations of the previously identified themes.

The identification of themes, patterns and negative cases was validated by one other member of the study steering group who read all the transcripts. After the first four interviews, an interim descriptive account based on the analysis described above was discussed with the steering group in order to validate emerging findings, the recruitment strategy and topic guide before continuing with further recruitment, interviews and analysis.

## Results

### Characteristics of interview participants

We approached a total of 54 local residents, of whom 12 (seven women and five men, aged between 34 and 72) completed an interview. Participants' homes were located in one of the four study neighbourhoods (Table 2) and lay between approximately 100 and 400 metres as the crow flies from the edge of the proposed new motorway (Figure 1). Six of the participants lived alone; five had access to a car; two currently cycled in the city; six were employed, one was disabled and five were retired. Each participant was assigned a unique identifier (P1 to P12) with which to annotate the results.

### Main themes elicited

**Figure 2:**
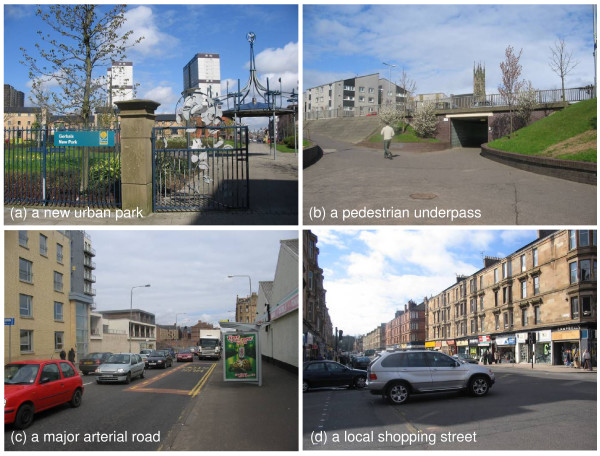
**Examples of scenes in and around the local study areas**. All images ^© ^David Ogilvie.

**Figure 3:**
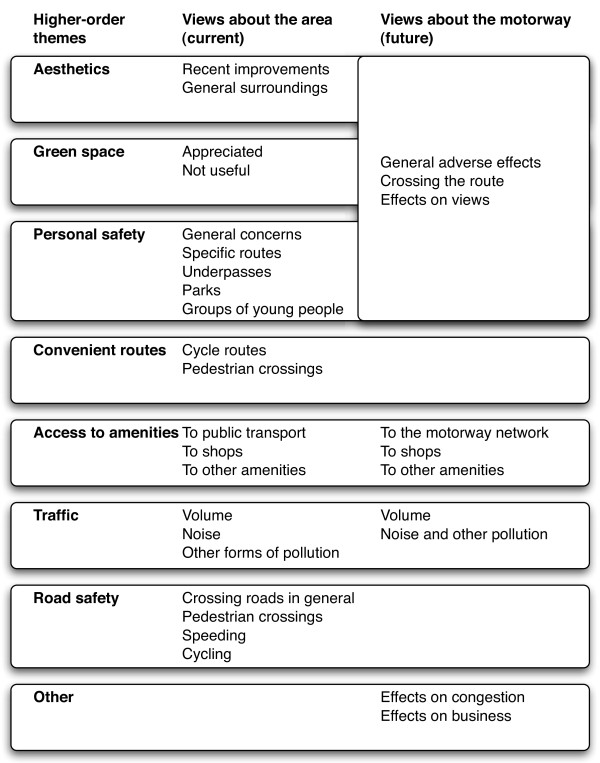
**Overview of key themes**.

Discussion of participants' local areas (Figure 2) elicited views related to all seven *a priori *environmental constructs, particularly access to amenities and road safety. All participants spontaneously mentioned the new motorway during their interview, and nine expressed views about its significance for the region as a whole. Discussion of the likely effects of the new motorway elicited views related to five of the seven *a priori *constructs; these were mostly related to traffic, access to amenities and aesthetics, with occasional instances related to green space and personal safety (Figure 3).

The content analysis is summarised below, with important themes and sub-themes illustrated by verbatim excerpts of transcripts. Dialect or colloquial terms are explained in square brackets, and local proper nouns are denoted by asterisks and explained in the glossary (Table 4).

**Table 4:** Glossary

Term	Definition
Glasgow Green; the Green	A large park adjacent to the River Clyde at the east end of the city centre
Kinning Park	Another suburb of Glasgow adjacent to the existing M8 motorway
Rouken Glen	A large park about 10 km away on the southern outskirts of Glasgow

### Views about the local area

#### Aesthetics

Two sub-themes emerged under this heading: recent improvements to the urban landscape, and the surroundings in general. Four participants praised recent improvements to a local high street (paving, lighting and so on) (P2, P5, P10) or open space (P9), although the improved open space was said to be used only by 'idiots on motorbikes' (P9) and not all participants believed that local people would be the main beneficiaries of recent improvements:

For me, it's no' for the people in Rutherglen, it's for the people that work in the place. And it's for people that come fae outwi' the toon [from outside the town] [...] So I think it's just been a kinda showpiece [...].but aye, it's lovely to look at (P5: man aged 51 with no car)

Most participants who commented on the general aesthetic quality of their surroundings were critical -- of graffiti, rubbish, smells, ugly buildings, or waste ground (P4, P6, P8):

It's not a particularly attractive area. Lots of graffiti around and things, it's not really a pleasant place, I mean you wouldn't go out just for a walk say. Unless it was for the good of your health (laughing). (P4: man aged 42 with car)

The exception was a woman who praised the tranquillity of her local area (P7), but unlike the others she had moved from one of Glasgow's notorious peripheral housing estates.

#### Green space

Views about green space were polarised. Some participants reported using parks as a cycle route -- 'sometimes I might cycle into town through the Green*' (P8) -- or as a place to meet friends (P8), take children to play (P2) or go for walks (P1, P3, P7), and others appreciated less formal local green spaces, including allotments, as a place to find tranquillity or for children to play (P2, P7). Others said local parks were too far away, unappealing, or had nothing to offer them or their families (P4, P5, P9, P10, P12):

Well, we're quite lucky here because we've got Queen's Park just up the road, down in the Gorbals we've got a lovely wee rose garden. They've built a new park and new houses, the Gorbals Park (Figure 2a), which is lovely. (P11: woman aged 64 with no car)

There's certainly nothing there for thirteen- to seventeen-year olds, apart fae [from] the Buckie [Buckfast, an alcoholic drink sometimes consumed by young people in parks], I suppose [...] it's just kinda a wee bit far away, and I wouldnae [wouldn't] be dead comfortable wi' my thirteen-year-old saying she wanted to go there. There's a place up there they can go wi' their bikes as well, the old putting green, but that's never very busy. (P5: man aged 51 with no car)

### Convenient routes

Two sub-themes emerged under this heading: cycle routes and pedestrian crossings. Four participants (not all active cyclists themselves) described either a local example of a good off-road route for cycling (P1, P2, P8) or an improvement in on-road provision for cyclists (P11). Four mentioned that there were plenty of pedestrian crossings on major roads (P2, P4, P5, P7), but these were not necessarily used (P2, P5, P9) or located at the most convenient points to cross (P1, P5, P9). The requirement to use underpasses ('tunnels': Figure 2b) to cross a dual carriageway was seen as an inconvenient detour (P2, P5) -- particularly for those with limited mobility (P10) -- and a threat to both personal safety and road safety:

*Do you ever go in the underpasses in Rutherglen? *Eh, no. Never go in them [...] I'll take a long way round rather than go in an underpass [...] I know that there's young people been attacked, you know [...] I know that that goes on and I'm no' gonnae [not going to] look for trouble. (P7: woman aged 64 with no car)

Somebody's pulled away a section o' the fence [...] So everybody just tends to use it. I mean, you see mothers going up in the morning wi' their kids, taking them to school, and they take them across that as well [...] they'd rather just go straight across there than going the long way roond, doon [round, down] through the tunnels and up [...] I don't suppose they think o' the consequences if they've got their kids wi' them and if they got hit by a car. (P9: man aged 49 with no car)

### Access to amenities

Three sub-themes emerged under this heading: public transport, shops, and miscellaneous other amenities.

Access to public transport (particularly bus services) was generally regarded as good, services being described as nearby, frequent and offering a choice of routes (P1, P3, P4, P5, P7). However, two participants with limited mobility had found it impossible to use local public transport with their wheelchair or mobility scooter (P4, P10), and two women described how a lack of suitable public transport provided something of an incentive to walk instead (P6, P12):

I probably walk to work more than what I used to. And that's because of the lack of public transport [...] There's only one bus service - a very good bus service, and it's a regular bus service, but at peak times it's very, very busy. And it only goes one way. Whereas, I was in Bridgeton and they all went to town, but then one went this way, one went that way [...] and it says it's every ten minutes or something, and sometimes I have to disagree with that because you can sit at a bus stop for fifteen minutes and then of course, the next three buses that come along are full. (P6: woman aged 34 with car)

Five participants identified useful shops within walking distance of their homes (P1, P4, P7, P9, P11). In several interviews, however, it became clear that the mere presence of local shops was not a sufficient reason to use them: two participants preferred to shop in the city centre (P1, P4), one felt 'shut off' from the local shops which could only be reached via a pedestrian underpass (P10), and three considered their local shopping street to be in decline (P7, P8, P9). Views about other local amenities were similarly divided, as exemplified by two contrasting descriptions of the same local library:

We're lucky - the health centre's only down the road in --. Got a lovely library, you know, I do love the library. (P11: woman aged 64 with no car)

I don't go down that way, I mean I wouldn't dream of going, the -- library is down there but I don't use that, I use the -- library. Now isn't that strange. I've got a library on my doorstep but it's full of druggies [...] They've still never got rid of this element. (P1: woman aged 68 with no car)

### Traffic

Three sub-themes emerged under this heading: volume, noise, and other forms of pollution.

There was a widespread view that traffic was very heavy, with participants using terms such as 'horrendous', 'gridlocked' and 'constant flow' to describe the situation (P1, P2, P3, P4, P6, P7, P9, P11). On the other hand, one participant acknowledged that a new road constructed in the early 1980s had been beneficial in this regard (P5) and another drew a distinction between the traffic-calmed area immediately surrounding her home and her local area in general (P1):

I don't particularly like to walk on the main roads, for the simple reason of the traffic. Not that I'm afraid that I'll get knocked down. But it's the fumes. I can smell the diesel and it's not fresh air [...] *How has the traffic situation changed around here since you've lived here? *Much better in this part I live in because I'm off the main road [...] -- Street, which is out the back, is very congested, but it's a lot quieter here. (P1: woman aged 68 with no car)

Views about traffic noise were more evenly distributed: three participants described this in very negative terms (P10, P11, P12), while three others were not bothered by traffic noise, although in two cases this was dependent on having double glazing (P4, P6, P7). The impact of traffic noise was most powerfully illustrated by an account of an occasion when the traffic was temporarily stopped:

Last year, we'd a big burst water main at the Cross, so the traffic was all diverted. It was so peaceful. You wouldnae [wouldn't] believe it. You know, I went 'Oh, this is heaven, this is what it should be like.' [...] It was so peaceful, that, you know, it suddenly brought it home to you how much noise you were taking in every day. It was like being out in the country [...] And you just felt, oh, I could walk more here, you know? (P11: woman aged 64 with no car)

Three participants described disturbance from fumes, dirt or vibration, particularly one living on a main arterial road (Figure 2c):

The whole building would shake, it's constant, and it's like four or five in the morn [ing] [...] you were shocked out of your sleep, you know? [...] When my friends come up and they bring their babies, and it's hot in there, I can't open my front windows at all because I'm concerned about the pollution [...] You can tell by the window how much stoor [dust] and dust and grime comes off the traffic as well. (P12: woman aged 36 with no car)

#### Road safety

Four sub-themes emerged under this heading: crossing the road in general, pedestrian crossings in particular (see above), speeding and cycling.

Four participants mentioned the difficulty of crossing main roads in their local area, with one describing it as 'terrifying' (P12) and others identifying more specific problems such as 'dangerous' junctions or 'mad buses' (P1, P3, P11). Excessive speed was identified as a problem by four participants (P2, P5, P9, P11), but two others had no particular concerns in this regard (P1, P8). In contrast, cycling on the road was unanimously regarded as dangerous -- for adults as well as for children (P1, P2, P5, P6, P8, P12) -- and the only safe places identified to cycle were off-road (P1, P2):

When I was young, we used to go runs tae [to] Rouken Glen* and things like that, but the roads are just too busy noo [now]. You wouldnae want your weans [children] going oot on these busy roads noo. It's just locally, and maist [most] o' the time, just tell them to cycle on the pavement unless the roads are really quiet [...] I mean if, you see adults cycling to work, and they cycle doon [down] the pavement. You know? So if adults are wise enough, weans are just as wise. (P5: man aged 51 with no car)

#### Personal safety

Five sub-themes emerged under this heading: general concerns about personal safety, and the perceived danger associated with specific routes, underpasses and parks and with groups of young people hanging around.

Two participants considered their area to be generally safe (P5, P7), whereas two others described significant concerns with personal safety at night (P9, P12). Four participants (all women) identified particular routes or areas where they felt vulnerable on foot (P1, P3, P6, P12); two preferred to walk along busy roads for this reason (P3, P12), and for one of them, the perceived danger of walking along a quiet road was sufficient to persuade her to make a regular local journey by car:

*I was interested in what you were saying about going to the [railway] station, that you take your car to get there. Any particular reason why you do that? *Cos I'm always rushing in the morning (laughing) [...] And then I hate walking up that road. It's deserted in the mornings [...] I'll leave here about quarter past, twenty past seven, and there's never a soul around on that road, because there's no houses or anything on it, it just gives me the creeps. (P3: woman aged 47 with car)

Three participants mentioned concerns about personal safety in underpasses (P2, P7, P9; see above); another was unconcerned because he never went out at night (P10), while another was reassured by recent investment in lighting and closed-circuit camera surveillance (P5). Five participants mentioned concerns about personal safety in parks, as a result of which most preferred not to enter their local park at all (P3), alone (P7), on foot at night (P8), or without a dog (P12), or would not allow their children to go there unaccompanied (P5):

I'd went to Pollok Park last year, and I just felt unsafe. So many single, solitary people, that you don't know what they're really there for [...] So, I didn't feel comfortable, and I left quite quickly [...] I felt it once or twice in Queen's Park as well [...] I have had the dog with me on those occasions, actually, but I still felt uncomfortable and I've just left the region or the park altogether. (P12: woman aged 36 with no car)

Five participants (four women and one man) expressed concern about young people hanging about (P1, P3, P6, P7, P9). None recounted an actual incident in which they had been attacked or threatened by such a group, but the possibility of attack worried them:

You know, some of the young people are really fantastic, no harm in them at all but when you see them in a group with their hoods up and they're coming towards you, you just kinda hold your breath till you're by them, you know, cos you don't know whether they're gonnae [going to] bother you or no'. (P7: woman aged 64 with no car)

### Views about the new motorway

#### Strategic benefits

Two sub-themes emerged under this heading: effects on traffic congestion, and effects on business and employment. In addition, two participants commented that politicians had 'spun' the benefits of the new motorway to the local workforce (P5) or given out conflicting messages (P10; see also Table 1):

It's a fallacy to say that because you build a new factory round here, it's all gonna be local people that works in it [...] Employers will move into the area because it's convenient for them, no' convenient for the local work force. (P5: man aged 51 with no car).

Six participants (including both men and women and those with and without access to a car) envisaged a beneficial effect on traffic congestion, although these benefits were mostly described as applying to journeys starting or finishing outside Glasgow (P2, P3, P5, P10, P11) and not necessarily to the participants themselves or their local areas (P1, P3, P11). Two participants believed the motorway would have no effect on traffic congestion or would generate more traffic (P1, P8) and one simply concluded 'I really don't see any value to this' (P7).

Opinions about the effects on local businesses and employment prospects were mixed. One participant thought the new transport links would help local businesses (P4) and another felt confident that local factories relocated to make way for the motorway would be found alternative sites (P7). On the other hand, one participant thought local businesses would suffer from the loss of passing traffic (P10) and another considered it a 'fallacy' that the local population would necessarily secure jobs in new factories attracted to the area (P5).

#### Effects on access to amenities

Three sub-themes emerged under this heading: access to the motorway network, to shops, and to other amenities.

Four participants thought they, or members of their family, would benefit from quicker access to the motorway network for journeys beyond Glasgow (P2, P4, P6, P10). Two others commented that the existing motorway access points were not far away and that this benefit was 'definitely not worth it' (P3, P8).

Two participants welcomed the prospect of a new supermarket within walking distance, which they linked to the motorway development (P2, P7); another living close by considered this development unnecessary and likely to be ugly (P8).

One participant acknowledged the recent redevelopment of a play area, which he linked to the motorway development (P5). Other participants (mostly women) thought the new motorway would put them and others off walking to local amenities, even though the journey on foot would still be possible (P1, P8, P10, P11):

If that big motorway got built there, I wouldn't go near Victoria Road (Figure 2d), I'm sorry. Except to go to the doctor or dentist. (P1: woman aged 68 with no car)

I think if it was like that, I would certainly not be going up Govanhill anymore, no. (P11: woman aged 64 with no car)

#### Effects on traffic

Two sub-themes emerged under this heading: effects on the volume of traffic on local streets, and effects on pollution in the form of noise, fumes, dirt or vibration.

Four participants believed the new motorway would, or might, reduce the volume of traffic on local streets (P2, P7, P10, P12). Five others rejected this suggestion when it was put to them, arguing that local traffic was nothing to do with the motorway, that the motorway would encourage traffic growth, or that their neighbourhood would become 'boxed in' by traffic -- 'like living in a roundabout' (P1, P4, P5, P8, P11).

Five participants envisaged adverse effects from pollution: noise (P1, P8, P9, P11), fumes (P1, P10), dirt (P1), or vibration from lorries (P8). On the other hand, two participants (both men and both with access to a car) thought that the motorway would not result in a net increase in noise and that faster-moving motorway traffic would produce less pollution than existing queuing traffic on local streets (P2, P4). Another participant was reassured by the advice of a friend living close to a section of urban motorway elsewhere in the city:

Said to me, looking at the map, she says, you're no closer to the M74 as what she is to the M8 and she says she never hears it. So I thought, well, will it bother me? Probably not, no. (P6: woman aged 34 with car)

#### Effects on aesthetics, green space and personal safety

Three sub-themes emerged under this heading: general adverse effects, and two specific adverse effects: one concerned with crossing the route, the other concerned with the effect on views. For two participants, these concerns were sufficiently serious to motivate them to leave the area (P3, P5):

It will change the area. Quite, quite drastically I think [...] See years ago Rutherglen was one of these places where people really wanted to live. But not now [...] I just think people will not want to live here anymore, me being one of them, I have to admit [...] I'm trying to get my husband on my side now to move. (P3: woman aged 47 with car)

Four participants (all women) envisaged significant adverse aesthetic effects: that the motorway would be 'stark' (P12) or an 'eyesore' (P8), would generate smells (P1), would make walking 'pretty unpleasant' (P8) and would result in people not wanting to live in the area any more (P3):

When I visualise it, it makes me think of Kinning Park*, sort of a wee bit stark and big concrete, concrete everywhere. (P12: woman aged 36 with no car)

The two participants quoted previously as saying that they would no longer walk to their local amenities (both women of retirement age and without access to a car) explained their expectations by relating their experiences of walking under the existing M8 motorway at nearby Kingston:

I just wouldn't like to think that I would walk up there and this big motorway thundering over my head [...] With the thunder of that traffic, it's a bit scary [...] And the atmosphere. Congested. Polluted. (P1: woman aged 68 with no car)

Oh, it's horrible. If you have ever walked under it. Well it's just like slabs of concrete, isn't it? And you're hearing this traffic all the time [...] you know, it's quite scary. Just because it's dark. It's all dull [...] it's a cold feeling that all this traffic going on top of you [...] it's just a big cold, stark, concrete. It's just only built for cars. You know? Certainly not pleasant to walk under. (P11: woman aged 64 with no car)

One also associated this type of infrastructure with a higher risk of being attacked (P11).

Three participants commented that the new motorway would spoil their view of the city or the surrounding countryside (P5, P8, P9).

One participant objected to the taking away of a particular area of informal green space to provide for traffic (P12). On the other hand, two participants did not think the new motorway would affect the aesthetic quality of another such area because it was already adjacent to a main railway line (P2, P7).

## Discussion

### Interpretation

Six important overarching themes emerged from the thematic analysis summarised above: two in which we identified a high degree of consistency between participants' responses, one in which we identified a consistently heterogeneous set of responses, and three in which we identified something of a paradox.

#### The volume of urban traffic

Despite Glasgow's relatively low level of car ownership by UK standards, traffic in the city was widely perceived to be heavy. This finding complements that of our quantitative survey of perceptions of the local environment, in which traffic volume was the item most likely to be rated negatively by respondents [29]. However, we found no quantitative association between active travel and perceived traffic volume. Our interview data help to explain this null association. Although two of our interview participants described being deterred from walking along particular routes by heavy traffic, they did not describe being deterred from walking in principle, and their views were balanced by those of others who preferred to walk along busier roads, for example because they felt less vulnerable to personal attack. This suggests that the influence of traffic volume on walking is likely to vary between individuals and may primarily affect the choice of routes rather than the propensity to walk as such. This is not to deny that heavy traffic may have other, potentially more serious impacts on local residents, for example in terms of air and noise pollution, sleep disturbance and mental wellbeing.

#### The danger of urban cycling

In our quantitative survey, only 26% of the men and 18% of the women reported having access to a bicycle and only 1% of respondents reported any cycling on the previous day [29]. In some ways, the low level of cycling in our relatively deprived study population is somewhat surprising, because it is much cheaper to buy and run a bicycle than a car and a large proportion of journeys in urban areas are within a reasonable cycling distance. However, our findings are consistent with Parkin's observation that the propensity to cycle can no longer be assumed to be associated with not having access to a car; using 2001 census data for England and Wales, he found that cycling to work was more common in households with one car than in those with no car [33].

The low propensity to cycle in our study may also reflect our survey finding that road safety for cyclists was much more likely to be rated negatively than road safety for pedestrians, which is complemented by our interview data: cycling in the city was widely perceived to be dangerous for both adults and children, and favourable descriptions of contemporary urban cycling were largely confined to low-traffic or traffic-free situations such as Sunday mornings, off-road paths, or children playing on bicycles in quiet residential streets -- opportunities which may have limited relevance for the everyday, non-recreational use of bicycles. If the new motorway results in the diversion of some traffic from local streets, as has been claimed, then perceptions of road safety for cyclists on those streets may subsequently improve. However, this alone may not be sufficient to stimulate an increase in the use of bicycles: given the apparently widely held view that cycling on local streets is unacceptably dangerous, a more radical approach involving the provision of more traffic-free routes may be necessary in order to stimulate more cycling, particularly among young or novice cyclists.

#### The polarisation of views about the likely effects of the motorway

In contrast to the first two themes, participants' views about the likely effects of the new motorway were polarised on almost all aspects, including its strategic importance and its impacts on access to amenities, traffic and the environment in the local area. The polarity of these views was not obviously associated with participants' age, sex or household circumstances, except that concerns about short-range aesthetic and oppressive impacts of the new motorway infrastructure were raised only by women. The diversity of views elicited is not unexpected and reflects the range of positive and negative impacts claimed for the new motorway in the public discourse (Table 1). The anticipation of being 'boxed in' by traffic also echoes a description elicited in a previous study in Corkerhill, a suburb of Glasgow that had been severed by the new M77 motorway in the 1990s: 'You cannae [cannot] get out, you're suffocating, claustrophobic' [13]. Even if the motorway does reduce the volume of traffic on some main roads, traffic is likely to increase on other roads feeding the motorway junctions, and the public local inquiry found that the principles of environmental justice espoused by the government were likely to be breached because local residents would experience most of the adverse effects, while the benefits would mostly accrue to motorway users passing through from other, more affluent areas [25]. These findings therefore support the view, taken at the design stage of the study, that the follow-up phase should seek evidence of both beneficial and adverse impacts which may be inequitably distributed [4].

#### The paradox of proximity versus utility

The importance of proximity to amenities is a prominent theme in the literature on environmental correlates of physical activity, but our quantitative analysis found that environmental factors accounted for little of the variance in active travel or physical activity after demographic and socioeconomic characteristics were taken into account [29]. This is not an isolated finding, and debate continues as to whether such 'negative' findings represent a true lack of association, a failure to specify precisely which forms of physical activity are likely to be influenced by proximity to which amenities, or a failure to take account of the quality of those amenities as well as their proximity [34,35]. Our interview data show that people may choose not to use their local amenities, either because local parks, shops and libraries do not meet people's needs or are considered undesirable places to go, or because some of the benefits potentially associated with the new motorway, such as new sports facilities, new jobs or shorter journey times, are perceived by local residents as 'not for us', instead mainly benefiting people from outside the local area [36].

#### The paradox of incomplete severance

An important conclusion of the environmental impact assessment for the new motorway was that because it would largely follow an existing plane of severance (a main railway line) which had few existing crossing points, the journeys of local residents would be little affected by the new infrastructure [30]. This assumption is challenged by our interview data which suggest that the complete absence of a connection is not necessary for people to feel cut off from their surroundings; even where a pedestrian route is provided, some people may still experience severance, either because they have physical difficulty with using the route provided or because they perceive it to be unpleasant or unsafe [37]. The major concerns identified in this regard in our interviews were about underpasses (particularly where these involve steep inclines) and walking under viaducts carrying the new motorway over surface streets. Although the environmental impact assessment identifies the need to apply 'relevant standards of disabled access' and design measures (such as the adequate lighting of underbridges) to enhance the perception of personal safety, it deals more perfunctorily with 'other' ('perceived or psychological') forms of severance, such as the potential reconfiguration of residents' 'home territories' identified in previous studies [19-22]. Just as people may not necessarily choose to walk or cycle to amenities merely because they are local, they may also choose not to walk or cycle to them because the route, although technically passable, does not appeal to them.

#### The paradox of public transport and active travel

It is axiomatic in contemporary transport policy that providing high-quality public transport is important in promoting a shift away from car use and is likely to facilitate walking and cycling [38]. However, in our quantitative analysis we found no association between active travel and perceived access to public transport [29]. Our interview data suggest a possible explanation, which is that the influence of public transport on active travel depends critically on the context and, more specifically, on the other modes of transport with which public transport is competing. In populations with a high prevalence of car ownership, high-quality public transport may indeed provide an alternative to car use which enables a moderate quantity of walking or cycling to be included in journeys previously made entirely by car [39]. On the other hand, two of our participants with limited mobility had found themselves unable to use public transport, and two others (both women, able to walk and without access to a car) clearly described how an inadequate bus service acted as a stimulus to walk.

### Strengths and limitations of the study

The strengths of this study lie in our decision to combine quantitative and qualitative methods of inquiry and in our initial framing of the study to participants as being concerned with 'traffic and health in Glasgow' in general rather than with the motorway proposal in particular. The qualitative interview data help to explain some of the quantitative survey findings, add depth to our understanding of what life was like in the intervention study area prior to the arrival of the motorway, and contribute concrete illustrations of how the local urban environment may change and how this may affect local people. We did not mention the motorway proposal in our quantitative survey or when we solicited participation in the qualitative interviews -- partly to avoid discouraging survey responses from the control areas, but mainly to minimise the risk of our baseline data being biased by participants' prior knowledge of our specific hypotheses. As a result, the motorway proposal emerged naturally in the course of the interviews rather than as the defining topic from the start.

The main limitation of the interview study lies in its modest sample size and relatively low response rate, which raises -- as in the postal survey -- questions about the representativeness of the data elicited. For the interview study, the failure to recruit participants aged below the mid-30s is the primary concern in this regard, although the comparative unwillingness of this age group to participate in research is well-recognised [40], as is the more general problem of declining willingness to participate in research in deprived communities [41]. Despite the modest number of participants, however, we did elicit a wide range of views that appeared to mirror those raised in the public discourse about the new motorway (Table 1) and, in most cases, the replication of those views within the sample. To that extent, we believe have achieved the 'objective of reflect [ing] the diversity within a given population' 'rather than aspiring to statistical generalisability' [42].

Although the professional perspective of the first author and interviewer ('public health doctor') was declared to participants, the fact that he lived not far from the boundary of the study area was not. On the one hand, familiarity with the study area was undoubtedly helpful in recognising and understanding the locations and journeys described by participants. On the other hand, any public health researcher living in the vicinity would naturally be expected to have their own attitudes and beliefs about the controversial topic of the study. We therefore sought to ensure that participants discussed both positive and negative impacts of the new motorway, that interview data were also examined by another member of the study team with no such local connection, and that minority opinions were identified and reported.

## Conclusions

Our findings have both substantive and methodological implications.

The main substantive implication is that the presence (or the insertion) of particular amenities and structures in the built environment is likely to influence walking and cycling in ways that may vary between individuals, may be inequitable, and may not be predictable from quantitative spatial data alone. For example, an underpass beneath a major road that apparently provides a more direct, traffic-free route for pedestrians and cyclists to reach their local shops may be experienced by some -- particularly women, older people and those with limited mobility -- as a new cause of isolation and severance, whether physical (because they are physically unable to use the route) or psychological (because it is perceived as a threatening or unpleasant environment). Similarly, the amenity value of well-maintained open spaces such as parks in more affluent areas may not be replicable in other areas where parks may be more likely to be neglected, unlit, or perceived as places to be avoided for various reasons. This may help to explain why an increasing number of correlational studies have failed to find significant overall associations between active travel and relatively simple summary spatial characteristics, such as proximity to key amenities, that do not take account of the qualitative characteristics of the amenities or the routes to them [43]. It also supports recent evidence-based guidance which notes, for example, that new infrastructure for cycling has only been shown to be effective in promoting cycling when built and maintained to a high standard [44]. The important, if obvious, theoretical implication is that most current ecological models of behaviour, which typically list a large number of individual, social and environmental explanatory variables without really explaining how these interact, are likely to be insufficient for use in predicting or evaluating the effects of interventions in the built environment, particularly with respect to identifying inequitably distributed effects. The development of a more applied ecological model for this purpose is the subject of another paper (Ogilvie et al, submitted for publication).

The key methodological implication is that including even a modest piece of qualitative research in the baseline phase of an intervention study can generate explanations, insight and hypotheses that do not emerge from the main body of quantitative data. Quantitative and qualitative methods can be combined in various ways in mixed-method studies, and this study illustrates several of them: our qualitative data have helped to explain quantitative findings (included unexpected or null associations) concerning the relationships between active travel and traffic volume, proximity to amenities and access to public transport; have given voice to local residents' views, experiences and expectations that were not accessible through the analysis of more abstract, quantitative data; and have given rise to new hypotheses (such as that concerning the inequitable effects of psychological severance) to be explored further in longitudinal qualitative analysis and tested in longitudinal quantitative analysis.

## Competing interests

This paper is partly based on material contained in the PhD thesis of the first author, who lived near (but not in) the intervention study area during the period when the fieldwork was conducted.

## Authors' contributions

DO had the original idea for the study, designed the study, applied for ethical approval, conducted the interviews, cleaned and analysed the data and wrote the paper. MP was DO's PhD supervisor, read the transcripts and validated the coding and analysis. RM, NM, MP and SP constituted the steering group for the study, contributed to and advised on the design of the study and the interpretation of the emerging findings, and contributed to the critical revision of the paper. All authors read and approved the final version.
